# Seasonal Variation in Food Consumption, Assimilation, and Conversion Efficiency of Indian Bivoltine Hybrid Silkworm, *Bombyx mori*

**DOI:** 10.1673/031.012.8201

**Published:** 2012-07-13

**Authors:** V. K. Rahmathulla, H. M. Suresh

**Affiliations:** ^1^ Central Sericultural Research and Training Institute, Mysore, Central Silk Board, Karnataka, India 570 008; ^2^ Present address, P3 Basic Seed Farm, National Silkworm Seed Organization, Central Silk Board, Mysore, Karnataka, India

**Keywords:** ingesta, digesta, nutritional efficiency parameters, bivoltine silkworm

## Abstract

Food consumption and utilization is influenced by various biotic and abiotic factors. Under different environmental, feeding, and nutritional conditions, and with ingestion of the same amount of mulberry leaves, the silkworm shows significant difference in its ability to digest, absorb, and convert food to body matter. Here, influences of season, temperature, and humidity on food intake, assimilation, and conversion efficiency of the Indian bivoltine hybrid (CSR2 × CSR4) *Bombyx mori* L. (Lepidoptera: Bombycidae) were studied. The results indicated that food ingestion and assimilation were significantly higher among silkworm batches where optimum temperature and humidity were maintained compared with silkworm batches exposed to natural climatic conditions of the respective season. However, during summer the nutritional efficiency parameters were significantly higher among silkworms reared under natural temperature and humidity conditions when compared with the control. During the winter and rainy season, the nutritional efficiency parameters were significantly higher in control batches, where optimum temperature and humidity were maintained. Ingesta and digesta required to produce one gram of cocoon/shell were also lower in control batches for all seasons except summer. This may be due to the physiological adaptation of silkworms to overcome stress during the summer season.

## Introduction

Insects feed on a remarkably diverse number of organic substances. Even so, most species show a great degree of specificity in food selection and feeding. Competition and natural selection gradually drive and bind each insect species to a specialized food supply that it can utilize more efficiently than its competitors. The silkworm, *Bombyx mori* L. (Lepidoptera: Bombycidae) is a Poikilothermic insect and the main source for the production of silk. Environmental factors such as temperature, humidity, light, air, and feed quality and quantity have intimate influences on its growth and development. Food consumption and utilization are influenced by various biotic and abiotic factors (Reynolds and Nottingham 1985), of which the most important are atmospheric temperature and humidity at the time of rearing (Benchamin and Jolly 1984). In sericulture, food is a factor of paramount importance that regulates growth, development, and silk yield. Food intake and silk production in silkworms are very closely related to nutritional factors. Dietary efficiency of silkworms plays a major role in converting mulberry leaves consumed to silk. There are many contributing factors that determine the digestion and conversion efficiency of a breed; among these, eco—physiological condition and morphological deviation are major factors. Ingesting the same amount of mulberry leaves under different environmental, feeding, and nutritional conditions, the silkworm shows significant differences in its ability to digest, absorb, and convert to body matter.

Kafian (1982) demonstrated that evaluation of strains must be made on the basis of food utilization efficiency at different feeding amounts under favorable conditions for each sex. Sigematsu and Takeshita (1967) showed an increase in intake of mulberry leaves during late age with a decrease in rearing temperature. Effects of various environmental factors on nutritional and water requirements of insects, including the silkworm, have been well—studied by various workers (Hiratsuka 1920; Matsumara and Ishizuka 1929; Legay 1958; Ito 1978; Scriber and Slansky 1981; Naik and Delvi 1987; Junliang and Xiaffeng 1992; Mishra and Upadhaya 1995; Rahmathulla and Geetha Devi 2001). Ueda and Suzuki (1967) reported that physiological activities, food intake, and economic parameters were influenced by body temperature of the silkworm, increases in temperature (20–30 °C) resulted in a decrease of leaf—silk conversion rate. Shen (1986) reported high efficiency of conversion in silkworm larvae reared at low temperature. Muniraju et al. (1999) studied the effect of temperature on leaf—silk conversion and reported that low temperature (26 °C) throughout the rearing period favored higher silk conversion with better survival in the bivoltine silkworm. The present study was undertaken to test the hypothesis that there are effects of various environmental factors on food ingestion and assimilation, as well as silk production by *B. mori.*

## Materials and Methods

### Study location

**Figure 1.**
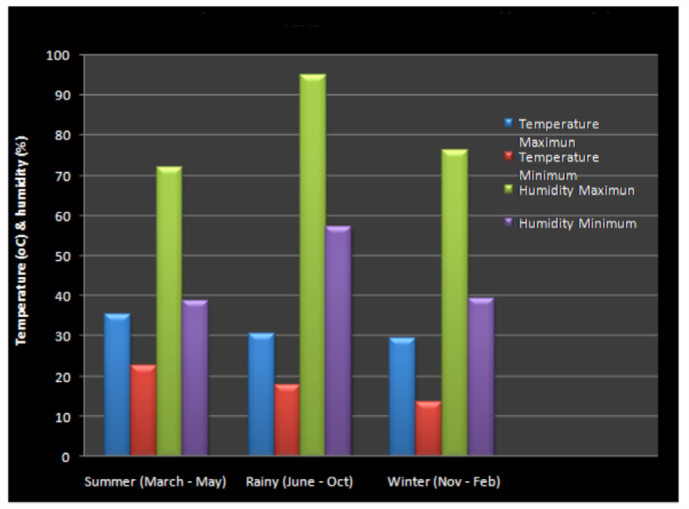
Effect of seasonal variation on temperature and humidity of the study location. High quality figures are available online.

**Figure 2.**
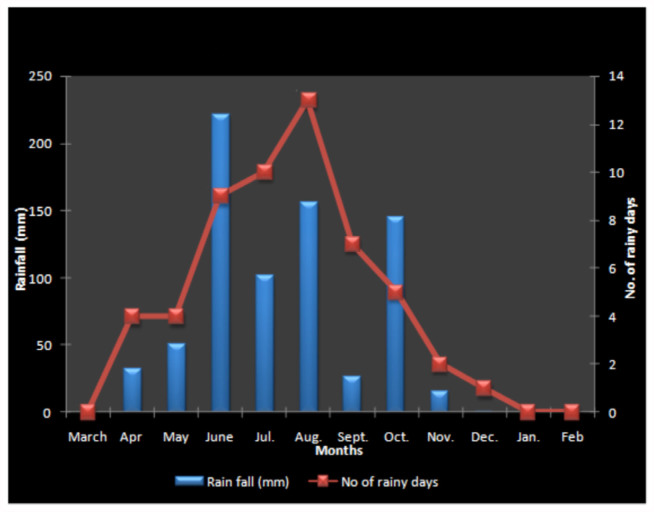
Rainfall data from the study location. High quality figures are available online.

The study area was located in Mysore, Karnataka, India at 12° 18′ N, 76° 39′ E, with an average altitude of 770 m. The area is located in the southern region of the state of Karnataka at the base of the famous Chamundi Hills, and spreads across an area of 128.42 km^2^. The summer season is from March to mid—June, followed by the monsoon season from mid—June to October, and the winter season from November to mid—February (Figure 1). The highest temperature recorded in Mysore was 38.5 °C, and winter temperatures as low as 9.6 °C have been recorded. The average annual rainfall received by the city is 798.2 mm (Figure 2).

### Study material

A productive and popular bivoltine hybrid silkworm (CSR2 × CSR4) developed by breeders of Central Sericultural Research and Training Institute, Mysore, India under the collaboration of Japanese subject experts was used in this experiment. The race is suitable to rear during more favorable seasons (August-February) under Indian environmental conditions. This hybrid is popular for its high survivability, high yield, high silk ratio, and its production of quality bivoltine silk matching international standards. Young silkworm rearing was conducted by following the new standard rearing package and practices (Rajan et al. 2001) by providing fresh tender leaves of V1 mulberry variety with high moisture content of 75–80%. Temperature (27 ± 1 °C) and humidity (85–90%) were maintained during young age rearing. During late age rearing (III and IV instar) the temperature was maintained 26 ± 1 ^°^C and humidity (70 ± 5%) up to fourth molt.

**Table 1.**
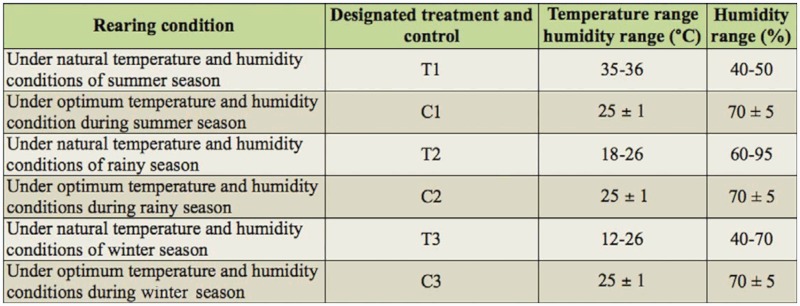
Details of the various treatments and control batches maintained in the study.

### Feed utilization studies

The feed utilization study was confined to the 5^th^ instar larvae, as 80–85% of the total leaf is consumed in this stage. The experiment was conducted during different seasons including summer, rainy, and winter. After the resumption from 4^th^ molt, 50 larvae each for three replications were separated and reared in natural conditions without maintaining temperature and humidity, and exposed to extreme temperature variation and high humidity conditions of the different seasons. For every season, a control batch with three replications of 50 larvae each was separated and reared in a sericatron—an environmental chamber with precise and automatic control facilities for uniform maintenance of temperature and humidity—and maintained at a temperature of 25 ± 1 °C and 70 ± 5% humidity. Known quantities of fresh quality mulberry leaves were provided to silkworms three times and followed the standard recommendation of the bivoltine rearing package (Rajan et al. 2001). The details of various treatments and control batches maintained during the present study are listed in Table 1.

Samples of mulberry leaves used for each feeding were placed in separate trays as dummies for dry weight determination of ingesta. Additional larval batches of each treatment were maintained in parallel to determine the dry weight and for subsequent determination of daily incremental changes in larval weight (Maynard and Loosli 1962). The healthy larvae were counted daily in each replication of every treatment, and unequal, unhealthy, and dead larvae were removed upon discovery and replaced from additional batches maintained separately. Observation on dry weight of leftover leaf, excreta, and larval weight were recorded daily after oven drying at a constant temperature of 80 °C. Larvae were mounted separately by replication and treatment in plastic, collapsible cocoon-— making frames (mountage). The cocoon harvesting and assessment was done after the 6^th^ day of mounting. The dry weight of cocoon and shell were also calculated. From the bioenergetics data, nutritional parameters like ingesta, digesta, approximate digestibility percentage (AD%), reference ratio (RR), consumption index (CI), conversion of ingesta and digesta (ECI and ECD) to larval body, cocoon, shell, and ingesta and digesta required to produce 1 g of cocoon and shell were determined by the standard gravimetric method described by Waldbauer (1968). The experiment was repeated two times in different seasons, and the pooled data were subjected to ANOVA to determine the significance. The formulas for calculation of different nutritional parameters are described below (Waldbauer 1968).

**Ingesta** = dry weight of leaf given — dry weight of leftover leaf

**Digesta** = dry weight of food ingested — dry weight of feces

**Consumption index** = ingesta / mean fresh larval weight × larval duration (days)

**Approximate digestibility percentage** (AD%) = (dry weight of food ingested — dry weight of feces / dry weight of food ingested) × 100

**Reference ratio** = dry weight of food ingested / dry weight of excreta

**ECI/ECD** to larva (%) = (dry weight gained by larvae during feeding period / dry weight of ingesta or digesta) × 100

**ECI/ECD** to cocoon (%) = (dry weight of cocoon / dry weight of food ingested or digested) × 100

**ECI/ECD** to cocoon shell (%) = (dry weight of cocoon shell / dry weight of food ingested or digested) × 100

**Ingesta required to produce 1 g of cocoon / cocoon shell** (ingesta / g cocoon / shell) = dry weight of ingesta / dry cocoon or cocoon shell weight

**Digesta required producing 1 g of cocoon / cocoon shell** (digesta / g cocoon / shell) = dry weight of digesta / dry cocoon or cocoon shell weight

## Results

### Nutritional indices parameters

Various nutritional indices are calculated and presented in Table 2. A significantly low value for ingesta was noticed in summer (T1: 3.85 g) when compared with the control (C1: 4.35 g). During the rainy season, significantly higher value of ingesta was recorded in control (C2: 4.72 g) when compared with natural climatic factors exposed batch (T2: 4.52 g). The same trend was observed in the winter season, and significantly higher ingesta was recorded for control batches (C3: 4.82 g) when compared with natural condition maintained batches.

**Table 2.**
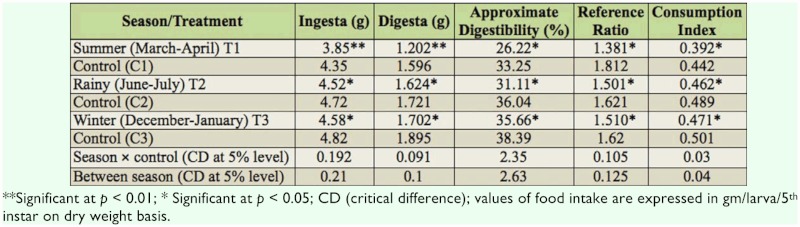
Influence of season on feed consumption and digestion of silkworm (*Bombyx mori*).

**Table 3.**
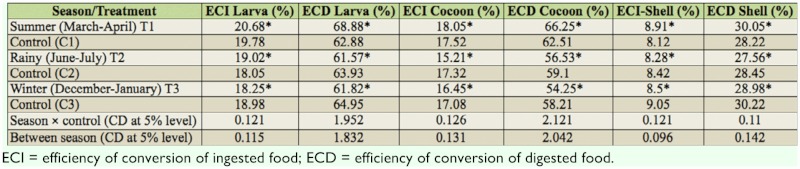
Influence of season on feed conversion efficiency of silkworm (*Bombyx mori*).

Similar to ingesta, digesta was also significantly higher in control batches when compared with respective natural temperature and humidity maintained batches. In summer, the lowest value of digesta was recorded in T1 (1.202 g) when compared with respective control (C1: 1.595 g). Significantly lower digesta was recorded in rainy (1.721 g) and winter (1.702 g) seasons when compared with respective control. Analysis by season showed that the maximum value of digesta was recorded during winter (T2: 1.721 g), and the lowest value was recorded in the summer (T1 : 1.202 g). Significantly lower approximate digestibility was recorded in summer (T1: 26.22%) when compared to respective control (C1: 33.25%). In other seasons, the same trend was observed, as significantly lower approximate digestibility was recorded in rainy (T2: 36.04%) and winter (T3: 35.66%) seasons when compared with respective control.

Significantly higher reference ratio (RR) values were recorded in control batches when compared with the seasonal batches (T1 : 1.381, T2: 1.501, T3: 1.510). Significantly higher consumption indices were recorded in control batches (C1: 0.442, C2: 0.489, C3: 0.501), where optimum conditions of temperature and humidity were maintained. Seasonal variation data indicated that during summer, the lowest consumption index was recorded (T1: 0.392).

### Nutritional efficiency parameters

Various nutritional efficiency parameters were studied and results are presented in Table 3. In the summer season, significantly higher ECI—larva was recorded (T1: 20.68%) when compared with the control (C1: 19.78%). However, in other seasons ECI—larva was recorded significantly higher in control batches (C2: 18.05%, C3: 18.98%), where optimum temperature and humidity was maintained. ECI—larva was recorded in T2 (19.02%) and T3 (18.25%) for rainy and winter seasons, respectively. Seasonal variation was noticed in ECI—larva, and the highest ECI—larva was recorded for summer, followed by rainy and winter seasons.

Similarly, ECD—larva was recorded as significantly higher during the summer season (T1: 68.88%) when compared with other two seasons. A significant difference of ECD—larva between control and normal batches was recorded during all seasons. ECD—larva (T1: 68.88%) was significantly higher when compared with control (C1: 62.88%) batch of summer. Significantly, lower values of ECD—larva, 61.57 and 61.82%, were recorded for rainy and winter seasons, respectively.

**Table 4.**
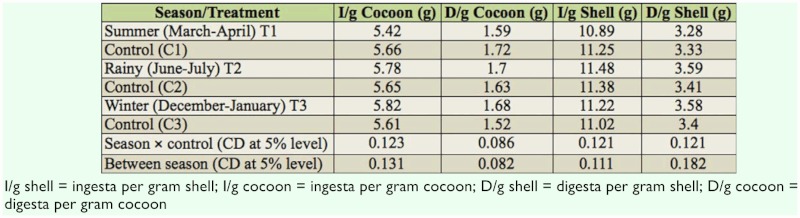
Influence of season on ingesta/digesta required to produce 1 g of cocoon/shell of bivoltine silkworm (*Bombyx mori*).

Significant difference of ECI—cocoon was observed between control and batches reared under natural climatic conditions. However, ECI—cocoon was significantly higher in summer—reared batches (18.05%) when compared to summer control (C1: 17.52%). In the rainy season, a significantly higher ECI—cocoon value of 17.32% was recorded for control batches when compared with the treated batches. Similarly, in winter, a significantly higher ECI—cocoon value (C3: 17.08%) was reported for control batches when compared with the treated batch (T3: 16.45%).

Similarly, ECD—cocoon was significantly higher in summer—reared batches (T1: 66.25%) when compared with the control (C1: 62.51%) and batches reared in the other two seasons. However, in the rainy and winter seasons, significantly higher ECD—cocoon was recorded in control (C2: 59.10%, C3: 58.21%) when compared with batches reared under natural conditions (T2: 56.53%, T3: 54.25%) in the rainy and winter seasons, respectively.

Significantly higher ECI—shell was recorded in batches reared under natural conditions of summer (T1: 8.91%) when compared with the control (8.12%) and batches reared in other seasons. However, in the rainy and winter seasons, significantly lower ECI—shell was recorded for batches reared under natural conditions (T2: 8.28% for rainy, T3: 8.5% for winter). Significant variation of ECD—shell was recorded among batches reared in different seasons. Similarly, higher ECD—shell (T1: 30.05%) was recorded for summer—reared batches when compared with batches of other seasons. In rainy and winter seasons, significantly lower ECD—shell was recorded in batches reared under natural conditions (T2: 27.56%, T3: 28.98%).

### Ingesta and digesta required to produce 1 g of cocoon and shell

Ingesta required to produce 1 g of cocoon was recorded significantly lower (5.42 g) in summer—reared batches when compared with corresponding control (5.66 g). Values of 5.78 and 5.82 g were recorded for rainy and winter seasons, respectively. Seasonal variation of data showed that in the summer season, the amount of food required to produce 1 g of cocoon was less than the other two seasons (Table 4).

The same trend was noticed in digesta required to produce 1 g of cocoon (D/g cocoon). This value was significantly lower in summer natural condition reared batches (T1: 1.59 g) when compared with the control (C1: 1.72 g), and it varied significantly when compared with other two seasons (1.70 g for rainy, 1.68 g for winter).

Ingesta required to produce 1 g of shell (I/g shell) was also significantly lower in summer natural condition reared batches (T1: 10.89 g) when compared with the summer control (C1: 11.25 g) and with batches of the other two seasons (T2: 11.48 g for rainy, T3: 11.22 g for winter). However, in the winter and rainy seasons, control batches had significantly lower I/g shell values.

The same trend was noticed in the case of D/g shell, with lower values for batches exposed to summer climatic conditions (T1: 3.28 g) when compared with summer control (C1: 3.33 g) and other batches. However, in the other two seasons, significantly higher D/g shell values were recorded in control batches for each respective season (C2: 3.41 g, C3: 3.40 g).

## Discussion

Most of the strains of bivoltine silkworm, due to consistent domestication, have become highly sensitive to variations in environmental conditions, especially seasonal variations in temperature and humidity in tropical parts of the Indian subcontinent. Fifth instar larval duration was significantly lower in the summer under natural environmental conditions compared with the control, where optimum temperature and humidity were maintained. Retarded growth in insects may be due not only to the nutritional inadequacy of the diet, but also due to the effect of various environmental conditions. Growth also depends on the digestion and utilization of food, which varies from species to species and even between different sexes of the same species. Further, reduction in feeding time in the most active feeding stage (5^th^ instar) in the bivoltine race, resulting in a shorter larval duration, adversely affected larval weight and other correlated traits.

Ingesta indicates the food ingested by the silkworm larva out of the food given. In the present study, analysis of seasonal influence on ingesta showed that the lowest and highest values for ingesta were in the summer and winter, respectively. Takeuchi et al. (1964) reported that there was an increase in intake of mulberry leaves during late age with a decrease in rearing temperature. Food consumption has a direct influence on the weight of larvae, cocoons, pupae, and cocoon shells. The independent parameters of consumption and productivity depend on the type of nutrition (Shivakumar 1995) and silkworm breeds (Remadevi et al. 1992). Digesta, which is the quantity of food assimilated, is affected by various factors. The enhanced digestibility, protease, and lipase activities indicate higher enzyme synthesis, corresponding to enhanced food intake. Similar to ingesta, significantly higher values of digesta were recorded for control batches.

Significantly higher values of approximate digestibility (AD%) were observed in all the control batches when compared with the respective batches reared in different seasons. Seasonal variation in AD% showed that winter season performance was comparatively better with a higher digestibility percentage. Higher values of AD% in control batches indicated the greater suitability of mulberry leaves and environmental conditions provided to silkworms. Since the digestibility differs in different treatments, the proportion of food intake and production of fecal matter also varied (Remadevi et al. 1992). Fecal matter values progressively increased as growth advanced; the production of excreta depends on availability of food, rate of food intake, absorption rate, and retention time of food in the gut. Mulberry leaves of superior quality and high water content directly regulate the phagostimulation, digestion, and efficiency of conversion in silkworms (Paul et al. 1992; Rahmathulla et al. 2004). Nutritional deficiency or imbalanced diet, high content of crude fiber, and deficiency of water in the food also affect digestibility.

Reference ratio (RR) is an indirect expression of absorption and assimilation of food. Higher RR values indicate high rate of digestion and absorption of food. The study results also found that comparatively higher RR values were recorded in control batches, and seasonal analysis shows that the maximum and minimum RR values were recorded in winter and summer, respectively. The consumption index (CI) is defined as the feeding rate at which nutrients enter into the digestive system. Trivedy and Nair (1999) suggested that CI was high when the rate of passage of food through the gut was rapid, allowing less time for digestion. On the contrary, when the passage of food through the gut was slow, the CI was high, allowing an increase in digestion and assimilation. In the present study, a higher CI was recorded in all the control batches, and summer climate exposed batches showed the lowest CI.

In a general entomological scenario, the nutritional efficiency of an insect may be considered an important parameter. In sericulture, however, nutritional efficiency becomes something of industrial importance. The dietary efficiency of silkworms can contribute considerably to the cost—benefit ratio of sericultural practice up to the level of cocoon production. The share of mulberry leaves accounts for more than 50% of the total cost of silkworm rearing. Different strains and hybrids of silkworm show significant differences in ability to digest, absorb, and convert mulberry leaves to body substance, and subsequently to the primary marketable product, the cocoon. The effect of different rearing seasons, planting density, rearing techniques, rearing conditions, quality of leaves, feeding proportion, and various chemicals such as food additives, vitamins, antibiotics, and hormones have an influence on the efficiency of conversion of food. In China, sericulture scientists have realized the importance of dietary efficiency, and they are engaged in finding effective ways to enhance leaf—silk conversion rate. This can be improved by breeding desirable silkworm strains and mulberry genotypes, providing good rearing environment and nutrition level, and by applying some insect growth regulators.

It is well known that food intake and silk production in silkworms is closely related to nutritional factors. Dietary efficiency of silkworms plays a major role in converting consumed mulberry leaves to silk. Efficiency of conversion of ingested (ECI) mulberry leaves into silk or leaf silk conversion rate is a better economic index in cocoon production. Trivedi and Nair (1998) also supported this statement, and these two parameters are the ultimate indices to evaluate the production efficiency of silkworm in terms of the production of cocoon shell percentage in relation to the food consumed (Maachi and Katagiri 1991). Ingesta and digesta are inversely proportional to efficiency of ingested and digested food to cocoon and shell. The efficiency of conversion of ingested food is an overall measure of the ability of larvae to utilize the ingested food. Leaf—silk conversion (ECI and ECD to shell) rate is composed of three basic factors, namely digestibility, the ratio of transforming digested food into cocoon and shell ratio.

In the present study, the feed conversion efficiency parameters such as ECI and ECD to larva, cocoon, and shell were significantly higher in control batches of the different seasons, except for summer, where all efficiency parameters connected with ingestion and digestion were significantly higher in batches exposed to natural weather conditions.

Many insects have adaptive behaviors to cope with stressful environmental conditions such as those present in the summer months; most of the feed conversion efficiency parameters were recorded significantly higher during this time period. Insects have evolved a variety of strategies to acquire and accumulate energy from nutrients and water available from food in a given environmental condition (Muthukrishnan et al. 1978). Singh and Ninagii (1995) reported that less food ingested and digested silkworm batches have high ECI and ECD to cocoon and shell. This may be because less choice of feed leads to some physiological adoptions to overcome nutritional stress (Nath et al. 1990; Tzenov 1993). Anantharaman et al. (1994) observed significant variation of ECI and ECD to larva when silkworms were fed with different varieties of mulberry leaves. Rahmathulla et al. (2004) observed that the efficiency of food ingested and converted to larval body matter varied prominently among different hybrids and under different feeding and environmental conditions. In the present study, results also confirmed that in the summer, batches reared under natural climatic conditions had a higher conversion efficiency of ingested and digested food.

Ingesta required to produce 1 g of cocoon was lower in summer—reared batches when compared with the corresponding control. This indicated that during the summer environmental conditions were unfavorable for silkworm growth, larval duration was shorter, the lifecycle was completed early and naturally, and less food was required to produce 1 g of cocoon. However, in the other two seasons, optimum temperature and humidity maintained (control) batches had lower I/g cocoon values when compared with batches reared under natural climatic conditions. In summer— reared batches, the lower ingesta and digesta required to produce 1 g cocoon and cocoon shell was due to its efficiency in converting the food under stressful conditions, which also depends on the genotypic character and its interaction with biotic and abiotic factors (Anantharaman et al. 1994). In the present study, less food was required under optimum temperature and humidity conditions to produce 1 g of shell when compared with the rainy and winter season batches.

Results show that the food ingestion and assimilation was significantly higher in optimum temperature and humidity maintained silkworm batches when compared with silkworm batches exposed to natural climatic conditions of respective seasons. However, nutritional efficiency parameters were higher in batches exposed to natural conditions of summer when compared with the control. During the winter and rainy seasons, nutritional efficiency parameters were significantly higher in control batches where optimum temperature and humidity were maintained. Ingesta and digesta required to produce 1 g of cocoon/shell were also recorded lower in control batches of all seasons except summer, which may be due to the physiological adaptation to overcome stress during the summer season.

For the last two decades, the introduction of productive bivoltine hybrids and rearing management in India has resulted in a phenomenal increase in yield and income level of the sericulture farmers, and has increased output of quality raw silk. However, these productive bivoltine hybrids can only provide large benefits to farmers who are able to provide high input and managerial skills. These hybrids lack genetic plasticity to cope with adverse temperature and humidity conditions of India. Therefore, it has become highly imperative to develop silkworm breeds tolerant to diseases and adverse climatic condition, or plan effective strategies for management of climatic conditions during silkworm rearing for better silk production.

## Figures and Tables

**Figure 3.**
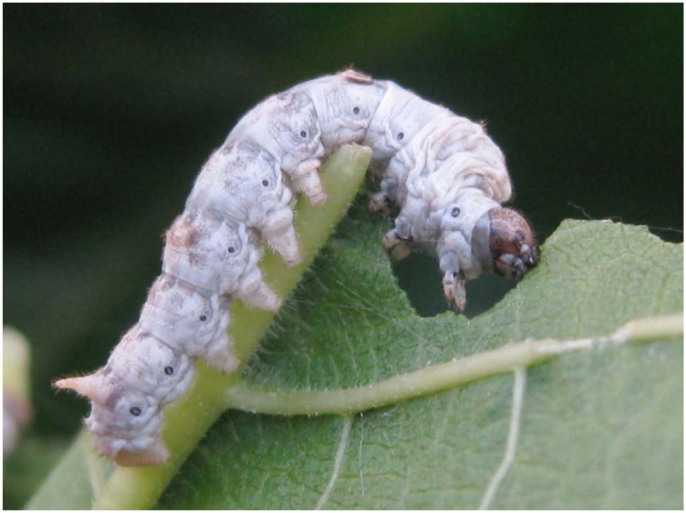
Fifth instar silkworm larva. High quality figures are available online.

**Figure 4.**
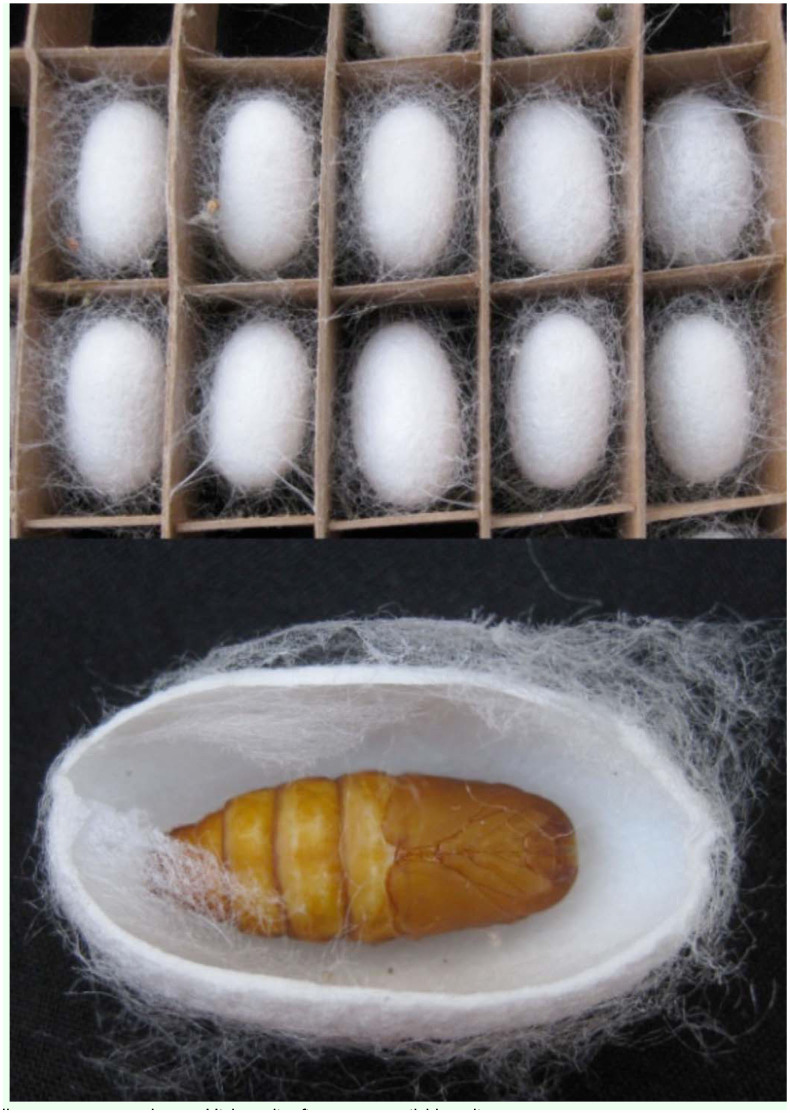
Silkworm cocoon and pupa. High quality figures are available online.
